# 

**Published:** 1883-01

**Authors:** 


					﻿Vol. IV.
JANUARY, 1883.
No. I.
THE
rnmtn Praehuoner:
yA yWoNTHLY jJoUF\NAL
DEVOTED TO
MEDICINE, SURGERY, OBSTETRICS, DENTISTRY, PATHOLOGY,
AND POPULAR SCIENCE.
EDITORS :
LEIGH EE. EETTITT, E.So., TA.ID.
VI. CL EABBETT, ID.ID.S., ZMZ.ID.
New York:
THE COLUMBIA PUBLISHING CO.,
42 DUANE STREET.
$2.50 Per Annum In Advance. -	- Single Copies, 25 Cents.
Entered at the Post-Office. New York City, as Second-Class matter.
				

